# Supplementary material for: Effects of Key Rumen Bacteria and Microbial Metabolites on Fatty Acid Deposition in Goat Muscle
Source: Animals (Basel). 2024 Nov 11;14(22):3225. doi: 10.3390/ani14223225 (PMC11590925; doi:10.3390/ani14223225)
Supplement: Supplementary file 1 [file animals-14-03225-s001.zip › Table S1- .pdf]

Table S1. Clustering turquoise module in rumen contents of Hechuan white goat correlates with the correlation analysis of five fatty acid content

ID	Metabolite	Trait	R	Pvalue
MEDN0245	Pantothenate	stearic_acid	0.8493462	0.000121
MEDN0385	FFA(12:0)	stearic_acid	0.7055309	0.004816
MEDN1055	D-Calcium Pantothenate	stearic_acid	0.7981962	0.000622
MEDN1946	Val-Asp	stearic_acid	0.8449394	0.000142
MEDN2122	Leu-Asp	stearic_acid	0.7382237	0.002571
MEDP0083	Trans-4-Hydroxy-L-Proline	stearic_acid	0.6988398	0.005423
MEDP0101	P-Coumaric Acid	stearic_acid	0.7159704	0.003977
MEDP0125	Choline	stearic_acid	0.7291041	0.003090
MEDP0246	Nicotinic Acid	stearic_acid	0.7458603	0.002192
MEDP0298	6-Aminocaproic Acid	stearic_acid	0.7339715	0.002804
MEDP0752	DL-Leucine	stearic_acid	0.7339715	0.002804
MEDP1072	7-Methylguanosine	stearic_acid	0.6749987	0.008082
MEDP1490	Cis-4-Hydroxy-D-Proline	stearic_acid	0.6988398	0.005423
MEDP1740	Acrylamide	stearic_acid	0.691766	0.006127
MEDP1988	Hydroxyurea	stearic_acid	0.7150659	0.004045
MEDP2002	Aminolevulinic acid	stearic_acid	0.6988398	0.005423
MEDP2068	Hexaethylene-glycol	stearic_acid	0.6873341	0.006604
MEDP2344	Cis-L-3-hydroxyproline	stearic_acid	0.6988398	0.005423
MEDP2388	Tyr-Asp	stearic_acid	0.6656909	0.009358
MEDP2391	Tyr-Glu	stearic_acid	0.7581998	0.001675
MEDP2421	1-Methylguanosine	stearic_acid	0.6749987	0.008082
MEDP2426	Proline-Hydroxyproline	stearic_acid	0.679471	0.007519
MEDP2529	D-Allo-Isoleucine	stearic_acid	0.8468117	0.000133
MEDN0245	Pantothenate	Palmitoic_acid	-0.720040	0.003683
MEDN0381	FFA(16:0)	Palmitoic_acid	-0.677739	0.007734
MEDN0385	FFA(12:0)	Palmitoic_acid	-0.805781	0.000503
MEDN0496	N-Acetyl-L-methionine	Palmitoic_acid	-0.668357	0.008978
MEDN0533	Xanthosine	Palmitoic_acid	-0.705001	0.004862
MEDN0662	Glu-Leu	Palmitoic_acid	-0.661771	0.009940

MEDN1055	D-Calcium Pantothenate	Palmitoic_acid	-0.668074	0.009018
MEDN1093	2-Isopropylmalate	Palmitoic_acid	-0.717739	0.003847
MEDN1946	Val-Asp	Palmitoic_acid	-0.683049	0.007091
MEDP0083	Trans-4-Hydroxy-L-Proline	Palmitoic_acid	-0.698234	0.005481
MEDP0101	P-Coumaric Acid	Palmitoic_acid	-0.766866	0.001373
MEDP0156	5-Methyluridine	Palmitoic_acid	-0.779686	0.001008
MEDP0171	Inosine	Palmitoic_acid	-0.740356	0.002461
MEDP0298	6-Aminocaproic Acid	Palmitoic_acid	-0.799364	0.000602
MEDP0461	2-Methylguanosine	Palmitoic_acid	-0.719232	0.003740
MEDP0507	2-Hydroxycinnamic acid	Palmitoic_acid	-0.745021	0.002232
MEDP0752	DL-Leucine	Palmitoic_acid	-0.799364	0.000602
MEDP0792	Pyrrolidine	Palmitoic_acid	-0.664110	0.009590
MEDP0821	6-Methylnicotinamide	Palmitoic_acid	-0.720195	0.003673
MEDP1072	7-Methylguanosine	Palmitoic_acid	-0.776132	0.001100
MEDP1093	Allopurinol	Palmitoic_acid	-0.704999	0.004863
MEDP1144	L-Isoleucine	Palmitoic_acid	-0.704336	0.004921
MEDP1193	Flavin Single Nucleotide(FMN)	Palmitoic_acid	-0.680613	0.007381
MEDP1490	Cis-4-Hydroxy-D-Proline	Palmitoic_acid	-0.698234	0.005481
MEDP1719	2'-O-Methylguanosine	Palmitoic_acid	-0.673918	0.008223
MEDP1740	Acrylamide	Palmitoic_acid	-0.682943	0.007104
MEDP1988	Hydroxyurea	Palmitoic_acid	-0.782408	0.000941
MEDP2002	Aminolevulinic acid	Palmitoic_acid	-0.698234	0.005481
MEDP2344	Cis-L-3-hydroxyproline	Palmitoic_acid	-0.698234	0.005481
MEDP2391	Tyr-Glu	Palmitoic_acid	-0.687199	0.006619
MEDP2408	3-aminobenzamide	Palmitoic_acid	-0.739743	0.002492
MEDP2421	1-Methylguanosine	Palmitoic_acid	-0.776132	0.001100
MEDP2529	D-Allo-Isoleucine	Palmitoic_acid	-0.867303	0.000059
MEDN1880	Hydroferulic acid	palmitic_acid	-0.751640	0.001936
MEDP2323	Diethyl-phosphate	palmitic_acid	-0.672321	0.008435
MEDN0062	N-Propionylglycine	oleic_acid	-0.665660	0.009363
MEDN1935	D-ribonate lithium salt	oleic_acid	-0.725659	0.003306
MEDP0156	5-Methyluridine	oleic_acid	-0.669832	0.008773

MEDP1509	Glu-Met	oleic_acid	-0.704629	0.004895
MEDN1880	Hydroferulic acid	myristic_acid	-0.723067	0.003476
MEDN1501	2'-O-methyluridine	Dh-γ-linolenic acid	0.708765	0.004543
MEDN0398	AA	Dh-γ-linolenic acid	0.678296	0.007664
MEDN0434	B-Pseudouridine	Dh-γ-linolenic acid	0.696818	0.005617
MEDN0506	N-Acetylglucosamine 1-Phosphate	Dh-γ-linolenic acid	0.679640	0.007499
MEDN0662	Glu-Leu	Dh-γ-linolenic acid	0.696177	0.005680
MEDN1302	D-Inositol 1,4-diphosphate	Dh-γ-linolenic acid	0.684337	0.006942
MEDN1506	3-Methyluridine	Dh-γ-linolenic acid	0.842229	0.000157
MEDN1606	Acetanilide	Dh-γ-linolenic acid	0.686571	0.006689
MEDN1620	Glu-Val	Dh-γ-linolenic acid	0.771550	0.001229
MEDN2135	γ-Glu-Met	Dh-γ-linolenic acid	0.766900	0.001372
MEDP0026	L-Valine	Dh-γ-linolenic acid	0.689562	0.006361
MEDP0156	5-Methyluridine	Dh-γ-linolenic acid	0.833861	0.000210
MEDP0170	Hypoxanthine	Dh-γ-linolenic acid	0.666366	0.009261
MEDP0171	Inosine	Dh-γ-linolenic acid	0.743220	0.002318
MEDP0298	6-Aminocaproic Acid	Dh-γ-linolenic acid	0.725233	0.003333

MEDP0380	2-(Dimethylamino)Guanosine	Dh-γ-linolenic acid	0.729301	0.003078
MEDP0752	DL-Leucine	Dh-γ-linolenic acid	0.725233	0.003333
MEDP0792	Pyrrolidine	Dh-γ-linolenic acid	0.699666	0.005345
MEDP0821	6-Methylnicotinamide	Dh-γ-linolenic acid	0.826757	0.000266
MEDP1072	7-Methylguanosine	Dh-γ-linolenic acid	0.687166	0.006622
MEDP1144	L-Isoleucine	Dh-γ-linolenic acid	0.763305	0.001491
MEDP1193	Flavin Single Nucleotide(FMN)	Dh-γ-linolenic acid	0.737474	0.002611
MEDP1472	N'-Methyl-2-pyridone-5-carboxamide	Dh-γ-linolenic acid	0.697446	0.005557
MEDP1509	Glu-Met	Dh-γ-linolenic acid	0.663997	0.009606
MEDP1511	Met-Glu	Dh-γ-linolenic acid	0.711849	0.004294
MEDP1719	Tetradecyl phosphonic acid	Dh-γ-linolenic acid	0.704615	0.004896
MEDP1778	L-Norvaline	Dh-γ-linolenic acid	0.689562	0.006361
MEDP1991	N6-Isopentenyladenosine	Dh-γ-linolenic acid	0.702146	0.005116
MEDP2131	L-Isserine	Dh-γ-linolenic acid	0.688492	0.006477
MEDP2408	3-aminobenzamide	Dh-γ-linolenic acid	0.693425	0.005956
MEDP2421	1-Methylguanosine	Dh-γ-linolenic acid	0.687166	0.006622

MEDP2529	D-Allo-Isoleucine	Dh-γ-linolenic acid	0.694038	0.005894
