# Supplementary material for: Effects of Key Rumen Bacteria and Microbial Metabolites on Fatty Acid Deposition in Goat Muscle
Source: Animals (Basel). 2024 Nov 11;14(22):3225. doi: 10.3390/ani14223225 (PMC11590925; doi:10.3390/ani14223225)
Supplement: Supplementary file 1 [file animals-14-03225-s001.zip › Table S2- .pdf]

Table S2. Sample grouping information

Grouping	Palmitoic_acid	Stearic_acid	Dh-γ-linolenic acid
HIGH	WHC2	Control1	Control1
	WHC5	Control4	Control2
	WHC1	Control5	Control5
	Control3	Control8	Control6
	Control9	WHC6	WHC6
LOW	WHC6	WHC1	Control3
	Control1	WHC2	Control4
	Control2	WHC3	Control8
	Control5	WHC5	WHC2
	Control8	Control6	WHC5
